# The Relationship of Dietary Nutrient Patterns and Irritable Bowel Syndrome: A Multicenter Case–Control Study

**DOI:** 10.1002/fsn3.70742

**Published:** 2025-08-19

**Authors:** E. Fazeli Moghadam, M. Samadi, S. Mohammadi, H. Yarizadeh, A. Abbasnezhad

**Affiliations:** ^1^ Nutritional Health Research Center, Nutrition, School of Health and Nutrition Lorestan University of Medical Sciences Khorramabad Iran; ^2^ Department of Nutrition, School of Nutrition Sciences and Food Technology Kermanshah University of Medical Sciences Kermanshah Iran; ^3^ Department of Community Nutrition, School of Nutritional Sciences and Dietetics Tehran University of Medical Sciences (TUMS) Tehran Iran; ^4^ Urmia Milad International Hospital Urmia Iran

**Keywords:** diet, irritable bowel syndrome, nutrient, nutrient patterns

## Abstract

Despite the connotations between some dietary patterns and irritable bowel syndrome (IBS), we are aware of no study about the link between IBS and patterns of nutrient intake. This study aimed to investigate the association between nutrient‐based dietary patterns and IBS in the Iranian adult population. This multicenter case–control study was conducted across three states in the west of Iran from 2021 to 2023. In this study, 317 patients with IBS and 601 healthy people were selected from the companions of other patients as a control group. Participants' dietary habits were evaluated using the validated and reliable 168‐item food frequency questionnaire (FFQ). The severity of extra‐intestinal IBS symptoms was measured using the Extra‐Intestinal Symptoms Severity Scale (EISSS). Three nutrient patterns were as follows: The first nutrient pattern was high in β‐carotene, vitamin C, vitamin A, α‐carotene, lutein, beta‐cryptoxanthin, lycopene, and vitamin E. The second nutrient pattern was rich in maltose, total fiber, glucose, and fructose. The third nutrient pattern included high consumption of sugars, sucrose, galactose, lactose, and caffeine. Logistic regression analysis after adjusting for the confounding variables showed that participants with higher adherence to pattern I (OR 0.75, 95% CI 0.62–0.92, *p* value 0.00) and pattern II (OR 0.52, 95% CI 0.42–0.64, *p* value 0.00) had lower odds of IBS. However, there was no significant connection between the third nutrient pattern and IBS (OR 0.95, 95% CI 0.80–1.12, *p* value > 0.05). Our study found that following nutrient patterns high in antioxidants, as well as those rich in monosaccharides and fiber, was primarily associated with lower odds of IBS.

## Graphical abstract

The current results found that following nutrient patterns high in antioxidants (β‐carotene, vitamin C, vitamin A, α‐carotene, lutein, beta‐cryptoxanthin, lycopene, and vitamin E), as well as those rich in monosaccharides (glucose and fructose) and fiber, were primarily associated with lower odds of IBS.

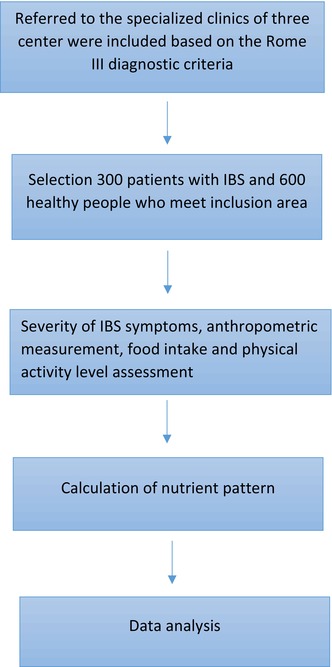

## 1 Introduction

Irritable bowel syndrome (IBS) is a common functional gastrointestinal disorder marked by symptoms including abdominal pain, bloating, and changes in bowel habits (Huang et al. 2023). Affecting a large segment of the global population, IBS presents a substantial burden on individual health and healthcare systems (Huang et al. 2023). Despite its prevalence, the underlying mechanisms and contributing factors remain poorly understood, prompting ongoing research into potential dietary influences (Carco et al. 2020).

Recent studies have begun to elucidate the role of nutrition in the prevention and control of IBS symptoms (Staudacher et al. 2020; Andrae et al. 2013). High intake of some specific nutrients such as vitamin C, β‐carotene, and caffeine might decrease the risk of IBS (Fuke et al. 2017; Pham et al. 2021). Nutrient patterns, which include the combined effects of various dietary components rather than focusing on isolated nutrients, provide a promising approach to understanding the relationship between diet and gastrointestinal health. For example, phytochemical compounds can provide gastro‐protective effects, for instance, by suppressing the inflammatory signaling pathways, ameliorating oxidative stress, and modulating gut microbiota (Lari et al. 2023).

Another dietary component that might be linked with IBS complications is the use of acidogenic dietary regimens. Preliminary evidence suggests that specific dietary patterns such as a diet low in fermentable oligosaccharides, disaccharides, monosaccharides, and polyols (FODMAPs), dietary total antioxidant capacity (DTAC), and dietary inflammatory index (DII) may influence gut microbiota composition, intestinal permeability, and inflammatory responses because of the high importance of these diets in IBS. Accordingly, it seems a low fermentable oligosaccharide, disaccharide, monosaccharide, and polyol diet may alleviate the symptoms of patients with IBS by reducing luminal acidity (Khalighi Sikaroudi et al. 2024; Mobasheri et al. 2022; Saneie et al. 2022). While we acknowledge that no research has specifically examined the relationship between dietary nutrient patterns and IBS, this manuscript aims to explore this connection.

By analyzing how different nutritional components influence the likelihood of developing IBS, we hope to offer valuable insights that could guide nutritional recommendations and therapeutic strategies for individuals at risk of or experiencing this complex disorder. Ultimately, this research seeks to enhance our understanding of the intricate connections between diet and gastrointestinal health.

## 2 Subjects and Methods

### 2.1 Study Design and Participant Enrollment

This multicenter case–control study was conducted in the years 2021–2023 at Lorestan University of Medical Sciences (city of Khorramabad), Urmia University of Medical Sciences, and Kermanshah University of Medical Sciences. In this study, 18‐ to 60‐year‐old IBS patients referred to the specialized clinics of each center were included based on the Rome III diagnostic criteria and with the approval of the gastroenterology subspecialist using the available sampling method. In the same way, healthy people were selected from the companions of other patients as a control group. The control and case subjects in the present study were collected at a ratio of approximately 2:1 from the central cities of three provinces: Khorramabad (197:103), Urmia (210:113), and Kermanshah (194:101). A total of 901 participants were involved in this study. Those with underlying diseases such as diabetes, liver and kidney dysfunction, cardiovascular disease, cancer, celiac disease, inflammatory bowel disease, and other digestive disorders; abdominal surgery; pregnancy; breastfeeding; having special diets; smoking; and alcohol consumption, and individuals currently undergoing treatment with gastrointestinal medications, drugs, or nutritional supplements were excluded from this study.

### 2.2 Data Collection

The Ethics Committee (IR. IUMS. REC.1399.308) of Lorestan University of Medical Sciences approved this cross‐sectional research, and informed consent was obtained from every participant. Two groups were matched for age and gender. The interviewers, who were proficient in questionnaires and measurement modules, then filled out the required questionnaires.

### 2.3 Anthropometric Measurements

Anthropometric measurements include height and weight, which were measured using a stadiometer with an accuracy of 0.1 cm and a digital scale with an accuracy of 100 g. The body mass index (BMI) is calculated by dividing the body weight (kg) by the height (m^2^) (Ramstrand et al. 2011).

### 2.4 Dietary Intake and Analysis

Dietary intake was assessed using the previously validated and reliable 168‐item Food Frequency Questionnaire (FFQ). This questionnaire, designed according to the Willette method, has a standard size for each food item.

First, during the face‐to‐face interview, the average size of each food item was explained, and then people were asked to report the frequency of consumption of each food item over the past year. Participants were asked about the frequency of food consumption based on the type of food they consumed daily, weekly, or monthly. The amounts reported by the subjects for each food item were converted to grams per day using the home comparison guidebook, and finally, the exact amounts of energy, micronutrients, and macronutrients consumed by each participant were calculated using the Nutritionist IV (N4) software (Mirmiran et al. 2010).

### 2.5 Severity of IBS Symptoms

The Extra‐Intestinal Symptoms Severity Scale (EISSS) was used to assess the severity of extra‐intestinal IBS symptoms. This instrument consists of 15 seven‐item questions (from never to always) that assess nonintestinal symptoms associated with IBS, including vomiting and nausea, early satiety, headache, back pain, fatigue, excessive flatulence, checking for excess, heartburn, urgency to defecate, feeling of incomplete bowel movement, urgency to urinate, thigh pain, muscle or joint pain, and feeling full after eating. The alternatives of sometimes to always experiencing the feeling were considered as the presence of a sign (Gholamrezaei et al. 2011).

### 2.6 Physical Activity Level

The physical activity of the participants was assessed using the International Physical Activity Questionnaire (IPAQ). This questionnaire collected information about physical activity during working hours, commuting, household chores, and free time during the past 7 days. Then, according to the standard instructions, the total metabolic equivalent score (MetS) was calculated, and the participants were classified into groups of low (up to 600 met/min/week), moderate (600 to < 3000 met/min/week), and high (at least 3000 met min/week) activity levels (Cleland et al. 2018).

### 2.7 Statistical Analysis

**FIGURE 1:**
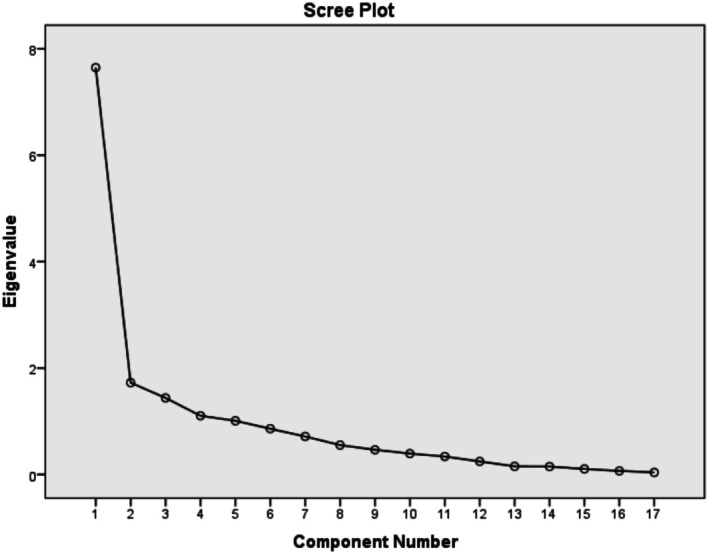
Scree plot of the nutrients and the extracted principal components in a case–control study on participants with irritable bowel syndrome (IBS).

The Kolmogorov–Smirnov test was used to assess the normality of the data distribution (β‐carotene, Vit.C, Vit.A, α‐carotene, lutein, beta‐cryptoxanthin, lycopene, Vit.E, maltose, total fiber, glucose, fructose, sugars, sucrose, galactose, lactose, caffeine nutrient patterns (I, II, and III)). To determine if the distribution of each nutrient variable was suitable for principal component analysis (PCA) and factor analysis, the Kaiser–Meyer–Olkin (KMO) test was conducted, applying a factor loading threshold of ≥ 0.4. Following this, PCA was performed, and the data were rotated using the Varimax method (Table 1). We also utilized the scree plot, focusing on Eigenvalues greater than or equal to 1.5, to identify the number of principal components (Figure 1). The analysis of qualitative variables to evaluate differences across nutrient pattern tertiles was conducted using cross‐tabulation and the Chi‐square test (Table 2). The differences in continuous variables across the tertiles of nutrient patterns were evaluated using one‐way analysis of variance (ANOVA, Tables 3 and 4). Fisher's LSD post hoc tests were used to compare tertiles of dietary patterns (Table 5). Binary logistic regression was used to examine the risk of IBS with nutrient patterns (Table 6). The significance level for all tests was established at a probability of ≤ 0.05. All statistical analyses were performed using IBM SPSS version 22.0 (SPSS, Chicago, IL, USA).

**TABLE 1:** Principal factor loading of nutrients intake.

Nutrient	Pattern I	Pattern II	Pattern III
β‐carotene	0.91		
Vit.C	0.89		
Vit.A	0.83		
α‐carotene	0.78		
Lutein	0.69		
Beta‐cryptoxanthin	0.68		
Lycopene	0.61		
Vit.E	0.58		
Maltose		0.91	
Total Fiber		0.89	
Glucose		0.56	
Fructose		0.48	
Sugars			0.78
Sucrose			0.74
Galactose			0.65
Lactose			0.51
Caffeine			0.43
Percent of variance explained	44.98	10.14	8.46

*Note:* Extraction method: principal component analysis; Rotation method: varimax with Kaiser normalization ^a^; a: rotation converged in 7 iterations.

**TABLE 2:** Number of participants across tertiles of major nutrient patterns.

Variables		Pattern I				Pattern II				Pattern III			
		T1	T2	T3	*p*	T1	T2	T3	*p*	T1	T2	T3	*p*
Gender	Male	135	137	143	0.41	142	136	137	0.96	120	140	155	0.05
	Female	131	132	113		129	120	127		137	123	116	
Group	Case	104	132	79	0.00	123	136	56	0.00	82	125	108	0.00
	Control	196	169	221		177	164	245		221	174	191	
Smoking	Smoker	98	125	81	0.00	111	99	94	0.00	92	109	103	0.61
	Non smoker	170	147	200		173	162	182		156	172	189	
	Quit smoking	7	5	0		10	0	2		5	5	2	
Education status	Illiterate	16	54	23	0.00	37	36	20	0.01	38	23	32	0.00
	Under diploma	25	30	27		24	22	36		30	27	25	
	Diploma	53	59	56		64	54	50		70	71	27	
	Educated	183	144	169		146	185	165		131	170	195	
Job status	Unemployed	72	92	80	0.07	77	94	73	0.01	92	68	84	0.00
	Housekeeper	128	115	126		136	106	127		98	133	138	
	Employee	79	68	43		54	70	66		61	79	50	
	Retired	7	10	7		13	11	0		24	0	0	
	Farmer	0	0	1		0	0	1		0	0	1	
Marital status	Single	127	182	159	0.00	162	157	149	0.62	170	154	144	0.10
	Married	161	97	123		120	136	125		112	137	132	
Physical activity levels	Light	45	27	32	0.00	24	44	36	0.03	30	39	35	0.02
	Medium	169	174	182		184	168	173		186	160	179	
	Heavy	16	26	39		35	20	26		18	22	41	

*Note:* *p* value obtained from Chi‐square test.

**TABLE 3:** Nutrient intakes across tertiles of main nutrient patterns.

Variables	Pattern I				Pattern II				Pattern III			
	T1	T2	T3	*p*	T1	T2	T3	*p*	T1	T2	T3	*p*
Energy intake (kcal/d)	2530.05 ± 793.76	2688.12 ± 797.39	2714.84 ± 1038.82	0.02	2474.77 ± 854.13	2486.65 ± 677.34	2970.66 ± 1007.21	0.00	2533.24 ± 783.76	2490.41 ± 829.58	2911.02 ± 978.14	0.00
Protein (gr/d)	72.18 ± 26.65	75.09 ± 23.82	79.78 ± 37.91	0.00	70.18 ± 33.00	72.25 ± 20.35	84.59 ± 33.43	0.00	73.93 ± 23.97	73.90 ± 34.59	79.25 ± 30.96	0.04
Carbohydrate (gr/d)	391.33 ± 134.15	40.9.94 ± 137.27	393.52 ± 157.36	0.22	362.14 ± 139.83	378.67 ± 116.41	453.83 ± 154.56	0.00	373.81 ± 124.12	381.57 ± 142.22	439.79 ± 153.72	0.00
Total fat (gr/d)	73.35 ± 27.79	83.60 ± 30.72	86.98 ± 39.21	0.00	80.65 ± 26.84	76.16 ± 29.27	87.10 ± 41.40	0.00	81.73 ± 30.1	74.17 ± 25.71	88.05 ± 41.16	0.00
Vit.A (mg/d)	208.73 ± 192.43	355.27 ± 278.58	853.68 ± 684.48	0.00	403.55 ± 481.79	337.55 ± 338.28	675.51 ± 632.78	0.00	485.47± 556.70	360.91 ± 364.1	570.75 ± 588.6	0.00
β‐carotene (mg/d)	973.28 ± 640.64	1910.95 ± 814.43	6482.59 ± 5902.28	0.00	3280.28 ± 4984.75	2079.77 ± 2569.19	3999.82 ± 4466.23	0.00	3452.45 ± 4567.60	2382.39± 2602.22	3523.52 ± 4974.33	0.00
α‐carotene (mg/d)	140.10 ± 158.27	234.26 ± 199.64	1195.13 ± 1580.99	0.00	388.83 ± 792.88	301.93 ± 445.49	876.60 ± 1490.43	0.00	412.44 ± 747.75	472.10 ± 1192.24	685.48± 1109.14	0.00
Lutein (mg/d)	679.04 ± 414.35	940.17 ± 458.63	1899.54 ± 1111.42	0.00	1163.12 ± 1049.63	973.46 ± 751.59	1380.72 ± 833.42	0.00	1244.06 ± 776.6	1010.92 ± 653.5	1262.05 ± 1169.26	0.00
Beta‐Kryptoxanthin (mg/d)	47.79 ± 48.14	64.89 ± 51.67	178.08 ± 181.82	0.00	86.65 ± 94.72	67.69 ± 99.58	136.19 ± 163.18	0.00	54.70 ± 69.91	84.53 ± 98.6	152.00 ± 169.07	0.00
Lycopene (mg/d)	1911.96 ± 1419.12	3095.50 ± 2109.61	6557.51 ± 4038.11	0.00	4253.40 ± 4405.77	3159.03 ± 2444.55	4149.03 ± 2887.14	0.00	2643.44± 2721.66	3678.67± 2745.03	5256.54 ± 4007.67	0.00
Vit.C (mg/d)	32.81 ± 19.54	61.96 ± 22.69	147.17 ± 79.84	0.00	89.65 ± 83.14	58.44 ± 41.01	93.77 ± 70.72	0.00	77.91 ± 81.33	69.13 ± 41.52	94.89 ± 75.09	0.00
Vit.E (mg/d)	7.63 ± 4.46	10.82 ± 4.05	14.82 ± 5.01	0.00	11.04 ± 6.06	9.76 ± 3.86	12.48 ± 5.66	0.00	9.17 ± 5.74	10.69 ± 4.21	13.45 ± 5.23	0.00
Total Fiber (gr/d)	93.72 ± 59.42	79.02 ± 37.09	107.08 ± 60.25	0.00	48.91 ± 28.78	79.10 ± 20.39	151.59 ± 45.69	0.00	89.53 ± 61.58	83.63 ± 43.07	106.67 ± 54.61	0.00
Sugars (gr/d)	82.35 ± 99.22	78.46 ± 54.36	99.62 ± 59.39	0.00	88.43 ± 103.00	71.67 ± 44.96	100.27 ± 59.48	0.00	44.13 ± 30.91	72.39 ± 32.91	144.46 ± 95.94	0.00
Glucose (gr/d)	13.45 ± 7.44	16.13 ± 12.05	23.57 ± 13.90	0.00	13.01 ± 7.96	14.30 ± 7.65	25.82 ± 15.07	0.00	17.09 ± 13.77	16.38 ± 10.42	19.70 ± 12.02	0.00
Galactose (gr/d)	2.43 ± 2.45	2.05 ± 1.69	2.90 ± 2.66	0.00	1.89 ± 1.98	2.29 ± 2.13	3.21 ± 2.65	0.00	1.12 ± 0.97	1.92 ± 1.24	4.37 ± 2.86	0.00
Fructose (gr/d)	9.64 ± 2.45	11.97 ± 7.70	18.92 ± 14.85	0.00	10.53 ± 9.12	10.80 ± 5.85	19.20 ± 14.87	0.00	8.18 ± 9.0	12.61 ± 7.27	19.83 ± 13.57	0.00
Sucrose (gr/d)	54.62 ± 82.14	53.03 ± 56.27	47.91 ± 30.71	0.36	59.05 ± 91.97	37.60 ± 27.08	58.90 ± 37.30	0.00	26.19 ± 17.7	44.14 ± 28.1	85.59 ± 89.16	0.00
Lactose (gr/d)	8.01 ± 7.61	8.66 ± 6.77	12.27 ± 8.96	0.00	7.85 ± 7.53	8.29 ± 6.25	12.80 ± 9.16	0.00	6.60 ± 6.18	7.35 ± 4.09	15.04 ± 9.8	0.00
Maltose (gr/d)	4.06 ± 2.49	3.10 ± 1.40	4.35 ± 2.84	0.00	1.86 ± 1.03	3.30 ± 0.93	6.35 ± 2.16	0.00	3.55 ± 2.4	3.39 ± 1.9	4.59 ± 2.50	0.00
Caffeine (mg/d)	131.87 ± 109.03	133.63 ± 100.76	133.98 ± 73.40	0.95	77.89 ± 4.49	99.40 ± 5.73	98.56 ± 5.68	0.00	68.10 ± 49.1	132.69 ± 76.1	199.56 ± 102.84	0.00

*Note:* *p* value obtained from ANOVA test.

**TABLE 4:** Components of demographics across tertiles of main nutrient patterns.

Variables	Pattern I				Pattern II				Pattern III			
	T1	T2	T3	*p*	T1	T2	T3	*p*	T1	T2	T3	*p*
Age (year)	31.09 ± 10.70	32.18 ± 10.91	30.63 ± 10.60	0.21	29.34 ± 8.52	33.01 ± 11.16	31.59 ± 12.02	0.00	29.68 ± 9.01	32.25 ± 11.51	31.96 ± 11.35	0.00
Body weight (kg)	72.64 ± 10.77	71.86 ± 11.76	75.75 ± 13.51	0.00	73.35 ± 13.18	73.88 ± 12.16	73.05 ± 11.14	0.70	73.37 ± 12.87	73.23 ± 11.78	73.66 ± 11.90	0.90
BMI (kg.m^−2^)	25.75 ± 3.70	24.90 ± 3.52	26.63 ± 4.15	0.00	26.00 ± 3.97	25.93 ± 3.92	25.35 ± 3.67	0.08	26.02 ± 7.27	25.95 ± 3.77	25.31 ± 3.46	0.04

*Note:* *p* value obtained from ANOVA test.

**TABLE 5:** The results of the one‐way ANOVA Fisher's LSD post hoc tests across tertiles of main nutrient patterns.

Variables	Pattern I *p* (MD)			Pattern II *p* (MD)			Pattern III *p* (MD)		
	*T1* vs. *T2*	*T1* vs. *T3*	*T2* vs. *T3*	*T1* vs. *T2*	*T1* vs. *T3*	*T2* vs. *T3*	*T1* vs. *T2*	*T1* vs. *T3*	*T2* vs. *T3*
Age (year)	0.22 (−1.08)	0.61 (0.45)	0.08 (1.54)	0.00 (−3.67)	0.01 (−2.25)	0.11 (1.42)	0.00 (−2.57)	0.01 (−2.28)	0.74 (0.29)
Body weight (kg)	0.43 (0.77)	0.00 (−3.10)	0.00 (−3.88)	0.59 (−0.53)	0.77 (0.29)	0.41 (0.82)	0.88 (0.14)	0.77 (−0.28)	0.66 (−0.43)
BMI (kg.m^−2^)	0.00 (0.85)	0.00 (−0.87)	0.00 (−1.73)	0.82 (0.07)	0.04 (0.65)	0.07 (0.57)	0.81 (0.07)	0.02 (0.71)	0.04 (0.63)

Abbreviation: MD, mean deffrence.

**TABLE 6:** Odds ratio (95% CI) for risk of irritable bowel syndrome based on main nutrient patterns.

	Variables	Pattern I			Pattern II			Pattern III		
Risk of IBS		OR	CI	*p*	OR	CI	*p*	OR	CI	*p*
	Crude	0.75	0.62–0.92	0.00	0.52	0.42–0.64	0.00	0.95	0.80–1.12	0.55
	Model 1	0.76	0.61–0.94	0.01	0.50	0.40–0.63	0.00	0.54	0.79–1.12	0.54
	Model 2	0.75	0.60–0.93	0.01	0.50	0.40–0.63	0.00	0.96	0.80–1.16	0.71
	Model 3	0.73	0.58–0.92	0.00	0.50	0.40–0.63	0.00	0.96	0.80–1.16	0.70
Severity of symptoms										
	Crude	1.02	0.85–1.22	0.82	0.03	0.67–0.98	0.03	0.91	0.77–1.08	0.32
	Model 1	1.04	0.86–1.25	0.67	0.80	0.66–0.97	0.20	0.90	0.76–1.08	0.28
	Model 2	1.11	0.91–1.35	0.28	0.85	0.69–1.04	0.12	0.99	0.82–1.20	0.96
	Model 3	1.11	0.91–1.36	0.29	0.85	0.69–1.04	0.12	0.98	0.81–1.20	0.91

*Note:* Binary logistic regression was used. Model 1: adjusted for age and gender. Model 2: additionally adjusted for total energy intake and physical activity. Model 3: additionally adjusted for marital status and medication.

Abbreviations: CI, confidence interval; IBS, irritable bowel syndrome, OR: odds ratio.

## 3 Results

### 3.1 Study Population Characteristics

The control and case subjects in the present study were collected at a ratio of approximately 2:1 from the central cities of three provinces: Khorramabad (197:103), Urmia (210:113), and Kermanshah (194:101). A total of 901 participants were involved in this study. The average age in the case and control groups was 29.72 (with a SD±10.19) and 32.14 (with a SD±10.91), respectively. The mean weight for the case and control groups was 72.66 (± 12.06) and 73.74 (± 12.24), respectively. In addition, the mean BMI for the case group was 25.84 Kg/m2 ± 3.87, while for the control group, it was 25.67 Kg/m2 ± 3.87.

### 3.2 Nutrient Pattern

Three nutrient patterns were discovered using principal component analysis (Table 1). The KMO value was 0.8, suggesting that the sampling adequacy was satisfactory. The first nutrient pattern was defined by elevated intake levels of Β‐carotene, vitamin C, vitamin A, α‐carotene, lutein, beta‐cryptoxanthin, lycopene, and vitamin E. The second nutrient pattern was rich in maltose, total fiber, glucose, and fructose. The third nutrient pattern included high consumption of sugars, sucrose, galactose, lactose, and caffeine. Overall, these three nutrient patterns explained 63.59% of the variance.

### 3.3 Qualitative Characteristics

Table 2 displays the association between the qualitative characteristics of participants and tertiles of discovered nutrient patterns. According to the chi‐square test, there was a significant relationship between the first nutrient pattern and smoking, physical activity levels, education status, and marital status (*p* < 0.05). A significant association was also found between the second nutrient pattern and smoking, physical activity levels, education status, and job status (*p* < 0.05). A significant correlation was identified between the third nutrient pattern and gender, physical activity levels, education status, and job status (*p* < 0.05).

### 3.4 Nutrient Intakes

The nutrient intake of participants across the tertiles of main nutrient patterns is presented in Table 3. According to the ANOVA test, there was a significant difference in all macronutrients and micronutrients (*p* < 0.05) across the categories of pattern I, except for sucrose (*p* = 0.36), caffeine (*p* = 0.95), and carbohydrates (*p* = 0.22). In addition, there was a significant difference in all macronutrients and micronutrients across the tertile of pattern II and pattern III (*p* < 0.05).

No significant differences were observed in age across the categories of pattern I. Moreover, subjects in the highest tertile of patterns II and III had higher age (*p* < 0.05) than those in the lowest tertile. Participants in the highest tertile of patterns I had higher weight (*p* < 0.05) than those in the lowest tertile. No significant differences were found in weight across the tertile of patterns II (*p* = 0.70) and III (*p* = 0.90). Our results show statistically significant differences in BMI among the categories of pattern I (*p* < 0.05) and pattern III (*p* < 0.05). However, these differences were not significant for BMI in the tertile of pattern II (*p* = 0.08). Table 5 shows the results of the one‐way ANOVA Fisher's LSD post hoc tests with age, weight, and BMI as the dependent variable and main nutrient patterns as the independent variable.

### 3.5 Risk of Irritable Bowel Syndrome

Logistic regression analysis showed that a reduced risk of IBS was observed in pattern I (OR 0.75, 95% CI 0.62–0.92, *p* value 0.00) and pattern II (OR 0.52, 95% CI 0.42–0.64, *p* value 0.00); even after adjusting for the confounder variables including age, gender, total energy intake, physical activity, marital status, and medication (Table 6). However, there was no significant relationship between the third nutrient pattern and the risk of irritable bowel syndrome (OR 0.95, 95% CI 0.80–1.12, *p* value > 0.05). We also found no significant relationship between the main nutrient patterns and the severity of disease symptoms.

## 4 Discussion

The current case–control study is the first to explore the link between dietary nutrient patterns and the risk of IBS. Our findings indicate that a higher adherence to nutrient pattern I, which includes substantial amounts of β‐carotene, vitamin C, vitamin A, α‐carotene, lutein, beta‐cryptoxanthin, lycopene, and vitamin E, as well as pattern II, which is characterized by elevated intakes of maltose, total fiber, glucose, and fructose, was associated with a reduced likelihood of developing IBS. This association remained significant even when controlling for variables such as age, gender, total energy consumption, physical activity, marital status, and medication. Conversely, no significant relationship was found between pattern III, which includes high levels of sugars, sucrose, galactose, lactose, and caffeine, and the risk of IBS.

Previous studies in this field have largely concentrated on dietary patterns like FODMAP (Mobasheri et al. 2022), dTAC (Saneie et al. 2022), and DII (Khalighi Sikaroudi et al. 2024) as well as the consumption of specific nutrients such as vitamin C (Pham et al. 2021), β‐carotene (Fuke et al. 2017), fructose (Choi et al. 2008), lactose (Varjú et al. 2019), and caffeine (Koochakpoor et al. 2021). However, there is a notable gap in understanding how nutrient consumption patterns interact with the risk of developing IBS. In the rapidly developing field of nutrition epidemiology, it is essential to explore how different nutrients interact with one another (Willett 2012). Our research aimed to explore the connection between different nutrient patterns and the risk of IBS. We identified three distinct nutrient patterns, each containing various complex nutrients. Patterns high in antioxidant vitamins and carotenoids were predominantly linked to a reduced risk of gastrointestinal diseases (Botterweck et al. 2000; Hengstermann et al. 2008). Additionally, patterns involving common monosaccharides and fiber were frequently associated with a lower incidence of gastrointestinal cancers (Slattery et al. 1988). Conversely, patterns dominated by sugars (including natural sugars from dairy) and caffeine have been associated with an increased risk of gastrointestinal disorders (Slattery et al. 1988).

Several studies have looked at the impact of individual nutrients on individuals with IBS. For example, one research study indicated that the combination of β‐carotene and probiotics improved gastrointestinal symptoms and reduced inflammation in IBS patients (Fuke et al. 2017). On the other hand, vitamin C has been found to influence intestinal inflammatory disorders by changing the gut microbiota (Ratajczak et al. 2020). An observational study conducted in Malaysia found that patients with IBS had a significantly lower intake of vitamin E compared to healthy individuals (Cai et al. 2024). Furthermore, consuming certain vitamins like vitamin A and β‐carotene can improve or sustain microbial diversity. In contrast, vitamin C is known to aid in producing short‐chain fatty acids, while vitamin E increases the abundance of microbes that produce these fatty acids, thereby impacting gut health and digestive functions (Pham et al. 2021).

Increased dietary fiber intake is known to alleviate symptoms and lower the risk of IBS (Currò 2022). Research indicates that individuals with IBS often experience malabsorption of fructose, leading to heightened gastrointestinal symptoms when their fructose consumption is high (Melchior et al. 2020). Interestingly, our study identified fructose as one of the micronutrients in dietary pattern II, which was linked to a decreased risk of IBS. For Iranians, the main source of fructose comes from fruits. Consumption of this food group, along with an overall increase in fiber intake, has been shown to positively impact IBS symptoms (Mobasheri et al. 2022). This may explain why dietary pattern II is characterized by high fiber content and reduced levels of fructose.

High levels of blood sugar and excessive sugar intake can disrupt the intestinal barrier, leading to increased gut permeability and notable alterations in gut microbiota (Arnone et al. 2022). Individuals with IBS often report issues with lactose and sugar intolerance (Dainese et al. 2014). However, it is not entirely clear whether these malabsorption symptoms stem from IBS itself or are due to lactose intolerance (Varjú et al. 2019). A meta‐analysis examining eight studies found that coffee consumption might be linked to a reduced risk of developing IBS (Lee et al. 2023). In contrast, another study revealed that caffeine intake was associated with a higher risk of IBS among women, but this connection did not apply to men. Additionally, a noteworthy correlation between caffeine consumption and IBS risk was seen in individuals with a body mass index (BMI) of 25 kg/m^2^ or more (Koochakpoor et al. 2021). Our study analyzed data from both genders and various weight categories—normal weight, overweight, and obese—which might clarify the lack of a significant relationship we observed between pattern III and IBS risk.

This study has multiple strengths, including the control for a broad array of potential confounding factors. Nevertheless, we cannot completely rule out the influence of residual confounders. We included only new cases of IBS, which minimizes the likelihood of alterations in typical dietary habits. We could not find a suitable model that includes fat, vitamin B group, vitamin D, vitamin K, and minerals, so we had to exclude them from the model. Our findings may be influenced by selection and recall biases that are typical in case–control studies. Like all epidemiological research, there is an unavoidable risk of misclassifying study participants due to the use of food frequency questionnaires (FFQs).

## 5 Conclusions

Our study found that following nutrient patterns high in antioxidants, as well as those rich in monosaccharides and fiber, was primarily associated with lower odds of IBS.

## Author Contributions

**S. Mohammadi:** data curation (equal), investigation (equal), methodology (equal). **A. Abbasnezhad:** investigation (equal), methodology (equal), project administration (equal).

## Ethics Statement

This study was approved by the Ethics Committee of Lorestan University of Medical Sciences (IR. IUMS. REC.1399.308).

## Conflicts of Interest

The authors declare no conflicts of interest.

## Data Availability

The data supporting this study's findings are available on request from the corresponding author. However, due to privacy or ethical restrictions, the data are not publicly available.
